# Egg parasitoid attraction toward induced plant volatiles is disrupted by a non-host herbivore attacking above or belowground plant organs

**DOI:** 10.3389/fpls.2014.00601

**Published:** 2014-11-05

**Authors:** Rihem Moujahed, Francesca Frati, Antonino Cusumano, Gianandrea Salerno, Eric Conti, Ezio Peri, Stefano Colazza

**Affiliations:** ^1^Dipartimento di Scienze Agrarie e Forestali, Università degli Studi di PalermoPalermo, Italy; ^2^Dipartimento di Scienze Agrarie, Alimentari e Ambientali, Università degli Studi di PerugiaPerugia, Italy

**Keywords:** *Trissolcus basalis*, Sitona lineatus, Nezara viridula, Vicia faba, indirect plant defenses, multi-trophic interactions, chemical ecology

## Abstract

Plants respond to insect oviposition by emission of oviposition-induced plant volatiles (OIPVs) which can recruit egg parasitoids of the attacking herbivore. To date, studies demonstrating egg parasitoid attraction to OIPVs have been carried out in tritrophic systems consisting of one species each of plant, herbivore host, and the associated egg parasitoid. Less attention has been given to plants experiencing multiple attacks by host and non-host herbivores that potentially could interfere with the recruitment of egg parasitoids as a result of modifications to the OIPV blend. Egg parasitoid attraction could also be influenced by the temporal dynamics of multiple infestations, when the same non-host herbivore damages different organs of the same plant species. In this scenario we investigated the responses of egg parasitoids to feeding and oviposition damage using a model system consisting of *Vicia faba*, the above-ground insect herbivore *Nezara viridula*, the above- and below-ground insect herbivore *Sitona lineatus*, and *Trissolcus basalis*, a natural enemy of *N. viridula*. We demonstrated that the non-host *S. lineatus* disrupts wasp attraction toward plant volatiles induced by the host *N. viridula*. Interestingly, *V. faba* damage inflicted by either adults (i.e., leaf-feeding) or larvae (i.e., root-feeding) of *S. lineatus*, had a similar disruptive effect on *T. basalis* host location, suggesting that a common interference mechanism might be involved. Neither naïve wasps or wasps with previous oviposition experience were attracted to plant volatiles induced by *N. viridula* when* V. faba* plants were concurrently infested with *S. lineatus* adults or larvae. Analysis of the volatile blends among healthy plants and above-ground treatments show significant differences in terms of whole volatile emissions. Our results demonstrate that induced plant responses caused by a non-host herbivore can disrupt the attraction of an egg parasitoid to a plant that is also infested with its hosts.

## INTRODUCTION

Parasitoids adopt specialized strategies to efficiently locate and parasitize their herbivorous hosts. Host-seeking females may exploit a plethora of cues, among which volatile organic compounds (VOCs) emitted by the plant as a consequence of herbivore attack, and therefore called herbivore induced plant volatiles (HIPVs), often play a key role (Kessler and Baldwin, 2001; Dicke, 2009). From the plant’s side, such a strategy developed as a consequence of plant-herbivore-parasitoid coevolution should be considered as an indirect defense against insect herbivores. It is known that plants defend themselves against herbivores either directly, through negative effects on herbivore performance, or indirectly, by recruiting natural enemies of the herbivore through synthesis and release of HIPVs (Kessler and Baldwin, 2001; Schoonhoven et al., 2005; Dicke, 2009; Ode, 2013). Numerous studies have documented the role of HIPVs as easily detectable and reliable host location cues for natural enemies of insect herbivores (Dicke and Baldwin, 2010; Kessler and Heil, 2011; Meiners and Peri, 2013). More recently, it has also been demonstrated that egg parasitoids exploit oviposition-induced plant volatiles (OIPVs; reviewed by Hilker and Meiners, 2010). Specifically, insect oviposition up-regulates plant defensive responses via the salicylic acid signal-transduction pathway (Bruessow et al., 2010; Reymond, 2013). Host eggs are unapparent, thus OIPVs provide female egg parasitoids highly detectable and reliable information on the presence of host eggs. Although OIPVs can recruit egg parasitoids of insect herbivores in some case studies, a combination of oviposition and feeding activity of the herbivore host is required to trigger attraction (reviewed by Colazza et al., 2010; Conti and Colazza, 2012).

Under natural conditions, plants often are attacked by multiple herbivore species, a scenario which potentially could interfere with the attraction of natural enemies as a result of modifications to the HIPVs blend (Soler et al., 2013). A growing body of evidence shows that communities and processes are intrinsically linked, and that they have important implications for community structure and ecosystem functioning (Dicke, 2009; Stam et al., 2014). These interactions may vary in terms of complexity and may involve organisms from several trophic levels and feeding guilds. Because we can expect plants to be adapted only to events that are common over evolutionary time spans, studies to elucidate these interactions preferably should be carried out at realistic densities and natural temporal sequences at which the various associations are established. To date, the land-mark studies demonstrating egg parasitoid attraction to OIPVs have been carried out in tritrophic systems consisting of one species each of plant, host herbivore, and egg parasitoid (Hilker and Meiners, 2002; Colazza et al., 2004a,b; Fatouros et al., 2012). Less attention has been given to plants experiencing multiple attacks by host and non-host herbivores that potentially could interfere with the attraction of egg parasitoids as a result of modifications of the OIPV blend. The interference effect on egg parasitoids could be affected by the non-host herbivore identity, feeding guild and impact on above- or below-ground plant tissues, as demonstrated for larval parasitoids (Rasmann and Turlings, 2007; Soler et al., 2007, 2013; Erb et al., 2010; Ponzio et al., 2014). We hypothesized that egg parasitoid attraction to induced plant volatiles could be influenced by temporal dynamics of multiple infestations, especially when the same non-host herbivore damages different organs, e.g. above- and below-ground, of the same plant species during the course of the growing season. We are not aware of any previous studies that have investigated the potential disrupting effect of a non-host herbivore, attacking either above- or below-ground plant organs, on attraction of egg parasitoids to volatiles produced by plants that are also infested with their typical hosts.

Therefore, in this paper, we investigate responses of the egg parasitoid *Trissolcus basalis* (Wollaston) to induced plant volatiles using the model system broad bean plants, *Vicia faba* L., infested with its typical host *Nezara viridula* (L.), and also infested with *Sitona lineatus* (L.), an above- and below-ground herbivore that is not a host for *T. basalis*. Previous investigations under tritrophic conditions have shown that the broad bean plant responds to *N*. *viridula* feeding and oviposition damage by emitting plant volatiles that recruit naïve *T. basalis* females. Such attraction was shown to be systemically induced and time specific, because feeding-damaged leaves bearing *N. viridula* eggs attracted the parasitoid until the eggs were ∼72–96 h old (Colazza et al., 2004a,b). In agro-ecosystems, broad bean plants can be attacked by over 50 herbivore species including aphids, leafhoppers, true bugs, thrips, moths, leafminers, and beetles (Bardner, 1983; van Emden et al., 1988; Nuessly et al., 2004). Among them, different life stages of weevils in the genus *Sitona* are known to attack different organs of the same plant throughout the growing season, and thus act as above- and below-ground herbivores. For example, adults of the pea weevil, *S. lineatus*, feed on foliage whereas larvae feed upon nitrogen-fixing bacteria *Rhizobium leguminosarum* Frank associated with rootlets and roots (Johnson and O’Keeffe, 1981; Hoebeke and Wheeler, 1985; Corre-Hellou and Crozat, 2005). In Italy, *V. faba* plants are commonly attacked by both *N. viridula* and *S. lineatus*, with adults of both species attacking above-ground plant parts early in growing season, whereas both above- and below-ground attacks occur later as the developing weevil larvae feed on the roots (Cusumano and Salerno, personal observations). To locate *N. viridula* eggs in such complex environments that undergo temporal changes in infestation by both hosts and non-hosts, and corresponding changes in plant-derived odor cues, *T. basalis* females could rely on learning abilities. In these circumstances, plasticity in a parasitoid’s response would be adaptive and learning could provide valuable flexibility (Peri et al., 2006; Fatouros et al., 2008; Colazza et al., 2010; Cusumano et al., 2012). Although it is well known that experience can strongly influence parasitoid foraging behavior, the role of previous experience in egg parasitoids foraging in a multitrophic system has not been addressed.

Thus, the aim of this paper was to investigate the effects of *S. lineatus* attack on the attraction of naïve and experienced females of the wasp *T. basalis* to *V. faba* plants that were being simultaneously attacked by the parasitoid’s host, *N. viridula*. Experiments were conducted with plants that were infested with insects above-ground, below-ground, and both above and below ground. The emission of plant volatiles also was evaluated in response to above-ground attacks under different experimental conditions.

## MATERIALS AND METHODS

### PLANT GROWING

Seeds of broad bean plants (*V. faba* cv. Superaguadulce) were immersed for 24 h in a slurry of water and soil (1:4) to favor root nodulation. The seeds then were individually planted in plastic pots (9 × 9 × 13 cm) filled with a mixture of agriperlite (Superlite, Gyproc Saint-Gobain, PPC Italia, Italy), vermiculite (Silver, Gyproc Saint-Gobain, PPC Italia, Italy), and sand (1:1:1) and grown in a climate controlled chamber (24 ± 2°C, 45 ± 10% RH, 12 h:12 h L:D). Plants were watered daily and, from 1 week post-germination, fertilized with an aqueous solution (1.4 g/l) of fertilizer (5-15-45, N-P-K, Plantfol, Valagro, Italy). In the case of “*above-ground treatments*” (see below), 18–20 days old broad bean plants, with approximately six fully expanded leaves, were used. For the “*below-ground treatments*” and “*above-* + *below-ground treatments,*” 15 days old plants were infested with *S. lineatus* eggs, left to grow to allow development of *S. lineatus* larvae on the root nodules, and then exposed to *N. viridula* (after 12 days) and/or tested (after 15 days; see below).

### INSECT REARING

The *N. viridula* colony established from material collected in cultivated and uncultivated fields around Perugia and Palermo (Italy), was reared under controlled conditions (24 ± 2°C; 70 ± 5% RH; 16 h:8 h L:D) in wooden cages (50 × 30 × 35 cm) with mesh-covered holes (5 cm diameter) for ventilation. Bugs were fed with a diet of sunflower seeds and seasonal fresh vegetables. Food was changed every 2–3 days, and separate cages were used for nymphs and adults. Egg masses were collected daily and used to maintain cultures of both *N. viridula* and *T. basalis.* The *N. viridula* colony was supplemented regularly with field-collected bugs.

*Sitona lineatus* adults were collected from *V. faba* fields around Perugia and Palermo and maintained in a climate-controlled chamber (8 ± 2°C; 70 ± 5% RH; 16 h:8 h L:D). The colony was reared in plastic food containers (30 × 19.5 × 25 cm) with 5 cm diameter mesh-covered holes. Adults were fed with vegetative parts of *V. faba* changed once a week and eggs were collected daily. Eggs were kept in Petri dish with the bottom covered by a filter paper disk moistened with distilled water. The Petri dish was sealed with Parafilm^®^ and maintained under controlled conditions (24 ± 2°C; 70 ± 5% RH; 16 h:8 h L:D).

The colony of *T. basalis* was originally established from wasps emerging from *N. viridula* egg masses, located in wild and uncultivated fields around Perugia and Palermo. The parasitoid was reared on *N. viridula* egg masses that were glued on paper strips. Wasps were maintained in 85 ml glass tubes, fed with a honey-water solution and kept in controlled environment room under the same rearing conditions of *N. viridula*. After emergence, male and female wasps were kept together to allow mating. For all bioassays, naïve or experienced (with oviposition experience on host eggs) 2–4 days old females were used. Naïve females were individually isolated in small vials 1 h before bioassays and then transferred to the bioassay room to be acclimatized. Experienced wasps were obtained with the following protocol: a *V. faba* leaf bearing a 24 h old *N. viridula* egg mass was placed in a circular arena (Ø = 1.8 cm; *h* = 0.5 cm), and then a single naïve *T. basalis* female was released in the arena to allow oviposition. After 10–15 min, experienced wasps (i.e., those that had parasitized one *N. viridula* egg) were recaptured and kept isolated in a small vial with a drop of honey-water solution for 24 h under controlled conditions (24 ± 2°C; 70 ± 5% RH; 16 h:8 h L:D) before being transferred to the bioassay room to be acclimatized for the next bioassay.

### PLANT TREATMENTS

**FIGURE 1:**
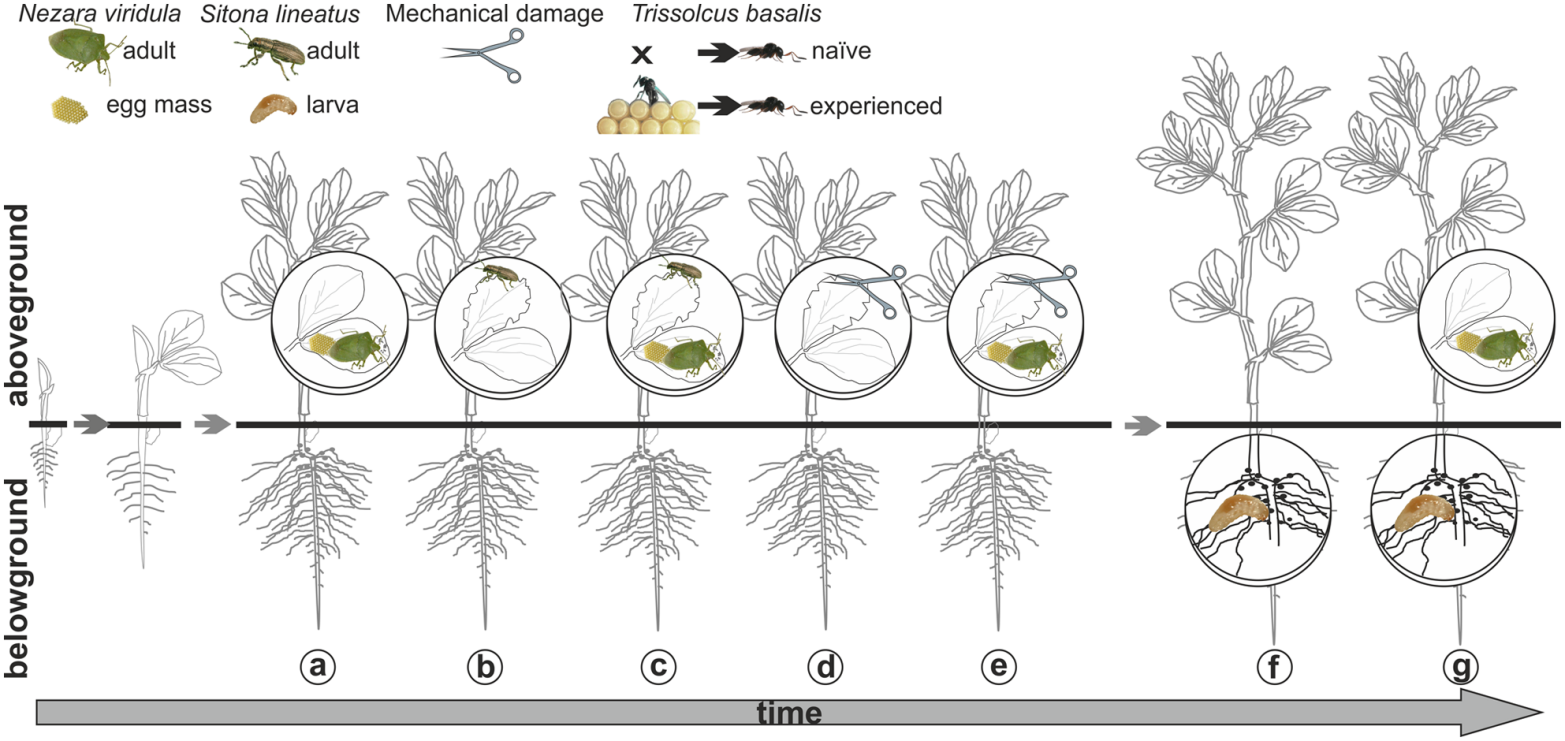
**Visual summary of the main plant treatments.** Above-ground *Nezara viridula* feeding and oviposition (a); *Sitona lineatus* adult leaf-feeding (b); *N. viridula* feeding and oviposition and *S. lineatus* adult leaf-feeding (c); mechanical damage (d); *N. viridula* feeding and oviposition and mechanical damage (e); below-ground (*S. lineatus* larvae root-nodules feeding) (f) and above + below-ground treatment (*N. viridula* feeding and oviposition and *S. lineatus* larvae root-nodules feeding) (g) and temporal dynamics of multi-trophic infestations on *Vicia faba.*

Plants were left untreated as controls, or subjected to the following treatments (**Figure 1**):

#### Above-ground damage

(a) *Nezara viridula* feeding and oviposition obtained by exposing individual plants to three *N. viridula* gravid females for 24 h.
(b) *Sitona lineatus* leaf-feeding obtained by exposing three leaves of a plant to 15 *S. lineatus* adults (five adults/one leaf) for 24 h using a “clip cage”; the latter consisted of two modified plastic Petri dishes (Ø = 10 cm; *h* = 1 cm), each with a mesh-covered hole in the bottom and the rim covered by a small sponge ring.
(c) *Nezara viridula* feeding and oviposition and *S. lineatus* adult leaf-feeding, obtained as described above by exposing the same plant first to *N. viridula* and then after 1 day to *S. lineatus* adults; attacks by the two species on the same leaves were avoided.
(d) Mechanical damage simulating *S. lineatus* leafdamage [leaf area removed by 15 adults in 24 h : 161.1 ± 27.45 mm^2^ (mean ± SE)], obtained by removing with scissors six triangular sections per leaf from three leaves (total leaf area removed: 132.88 ± 10.90 mm^2^).
(e) Mechanical damage combined with *N. viridula* feeding and oviposition. Plants were first exposed to *N. viridula* and, after 1 day, were damaged mechanically as described above; mechanical damage on the leaves carrying a *N. viridula* egg mass was avoided.

#### Belowground damage

(f) *Sitona lineatus* larvae feeding on root nodules, obtained by infesting individual plants with 30 *S. lineatus* eggs ready to hatch (6–7 days old). With the aid of a fine paintbrush, eggs were gently put inside a dimple made *ad hoc* on the plant substrate and then they were covered with the same substrate. These treated plants were tested 15 days after inoculation with eggs in order to allow larval feeding damage to the root nodules.

#### Above- + below-ground damage

(g) To get plants damaged with a combination of *N. viridula* feeding and oviposition and *S. lineatus* larval damage to root nodules, test plants were first infested with *S. lineatus* eggs as described above and, 12 days after inoculation, they were exposed to *N. viridula* as previously described.

### Y-TUBE OLFACTOMETER BIOASSAYS

Wasps’ responses to volatile chemicals from differently treated *V. faba* plants were investigated with a dual choice Y-tube olfactometer made from a polycarbonate body (stem 9 cm; arms 8 cm at 130° angle; ID 1.5 cm) sandwiched between two glass plates. A stream of clean air (medical-grade compressed air, N2:O2 80:20), humidified by bubbling through a water jar, was regulated in each arm by a flowmeter at about 0.4 l min^-1^. The device was illuminated from above by two 22-W cool white fluorescent tubes, and from below by an infrared source (homogeneous emission of wavelengths at 950 nm provided by 108 LEDs). Before entering the olfactometer arms, each air stream passed through a cylindrical glass chamber (Ø = 12 cm; *h* = 52 cm) with an O-ring sealed middle joint, containing a treated plant as odor source. The stimuli were randomly assigned at the beginning of the bioassays and were reversed after testing five parasitoid females. At every switch, the whole system was changed with cleaned parts. At the end of the bioassays the polycarbonate olfactometer and all glass parts were cleaned with water and detergent. The glass parts were then cleaned with acetone and baked overnight at 180°C. Wasp females were singly introduced into the Y-tube olfactometer at the entrance of the stem and allowed to move freely for 10 min. Their behavior was recorded using a monochrome CCD video camera (Sony SSC M370 CE) fitted with a 12.5–75 mm/F 1.8 zoom lens. The camera lens was covered with an infrared pass filter (Kodak Wratten filter 87 Å) to remove visible wavelengths. Analog video signals from the camera were digitized by a video frame grabber (Canopus^®^ ADVC 110, Grass Valley CA, USA). Digitized data were processed by XBug, a video tracking and motion analysis software (Colazza et al., 1999). Wasp response was measured in terms of residence time, i.e., the time spent by the wasps in each arm during the entire bioassay. The Y-tube olfactometer bioassays were carried out as paired choices, in which odor sources were always tested *versus* healthy plants used as control. Test odor sources included plants subjected to *above-ground, below-ground* and *above-* + *below-ground* treatments, described in the previous section. For each treatment, bioassays were conducted using either naïve or experienced wasp females. In the bioassays with *above-ground* and *below-* + *above-ground* treatments, the roots of each test plant were checked after the bioassay under a stereomicroscope to assess the presence of *S. lineatus* larvae and damaged root nodules. When no larvae were detected the data were discarded. About 40 replicates were conducted for each treatment. Bioassays were conducted from ∼09:00 to 13:00 h under controlled conditions (26 ± 1°C; 50 ± 5% R.H.).

## COLLECTION OF PLANT VOLATILES

A cylindrical glass chamber (Ø = 9 cm ID; h = 29 cm) was used to collect headspace volatiles from above-ground treated plants and healthy plant controls (*n* = 5 for each). Before each collection, the glass chamber was washed with water and detergent, rinsed with acetone, and baked overnight at 180°C. Singly potted plants were placed in each aeration chamber, separated from the pot and soil with two semi-circular Teflon plates to reduce contamination from soil odors. Air, purified by passage through an activated charcoal filter, was pumped into the chamber at 900 ml/min, with 600 ml/min being pulled through a glass tube filled with Porapak Q (Sigma Aldrich; 60 mg, 80–100 mesh), which was pre-cleaned with hexane and then heat conditioned for at least 2 h in a stream of nitrogen (100 ml/min) at 130°C. Volatiles were collected for 24 h, then traps were eluted with 700 μl of hexane, and the resulting extracts were concentrated to 100 μl under a gentle nitrogen stream. Extracts were stored at -20°C in glass vials with Teflon cap liners until used for gas chromatography (GC) analyses. For each plant used in the volatile capture, the total leaf area was measured.

## CHEMICAL ANALYSES

Gas chromatography-mass spectrometry (GC-MS) analyses were performed on a Hewlett-Packard 5890 GC system interfaced with an HP 5973 quadruple mass spectrometer. For each sample (*n* = 5), 1 μl of extract was injected onto a HP5-MS column (5% diphenyl–95% dimethylpolysiloxane 30 m × 0.2 mm, 0.25-μm film, J&W Scientific, Folsom CA, USA) in splitless mode. Injector and detector temperatures were 260°C and 280°C respectively. Helium was used as the carrier gas. The GC oven temperature program was 40°C for 5 min, then increased by 10°C/min to 250°C. Electron impact ionization spectra were obtained at 70 eV, recording mass spectra from 40 to 550 amu. Peak area of each detected compound was calculated and related to the total leaf area of the plant. The purpose of this chemical analysis was to investigate if the composition of the *V. faba* volatile blend varied according to the treatments; consequently no chemical characterization of the detected compounds was carried out.

## STATISTICAL ANALYSIS

For the bioassays, the time spent by wasp females in each arm was statistically compared by parametric paired *t*-tests for dependent samples and data were analyzed using the STATISTICA7 software (StatSoft, 2001). Data from analysis of volatiles extracts were analyzed by multivariate analysis using projection to latent structures discriminant analysis (PLS-DA) using the SIMCA-P+ 12.0 software program (Umetrics AB, Umeå, Sweden). This projection method determines if samples belonging to the different treatment groups can be separated on the basis of quantitative and qualitative differences in their volatile blends. The results of the analysis are visualized in score plots, which reveal the sample structure according to model components and loading plots, which display the contribution of the variables to these components as well as the relationships among the variables.

## RESULTS

### Y-TUBE OLFACTOMETER BIOASSAYS

#### Above-ground treatments

**FIGURE 2:**
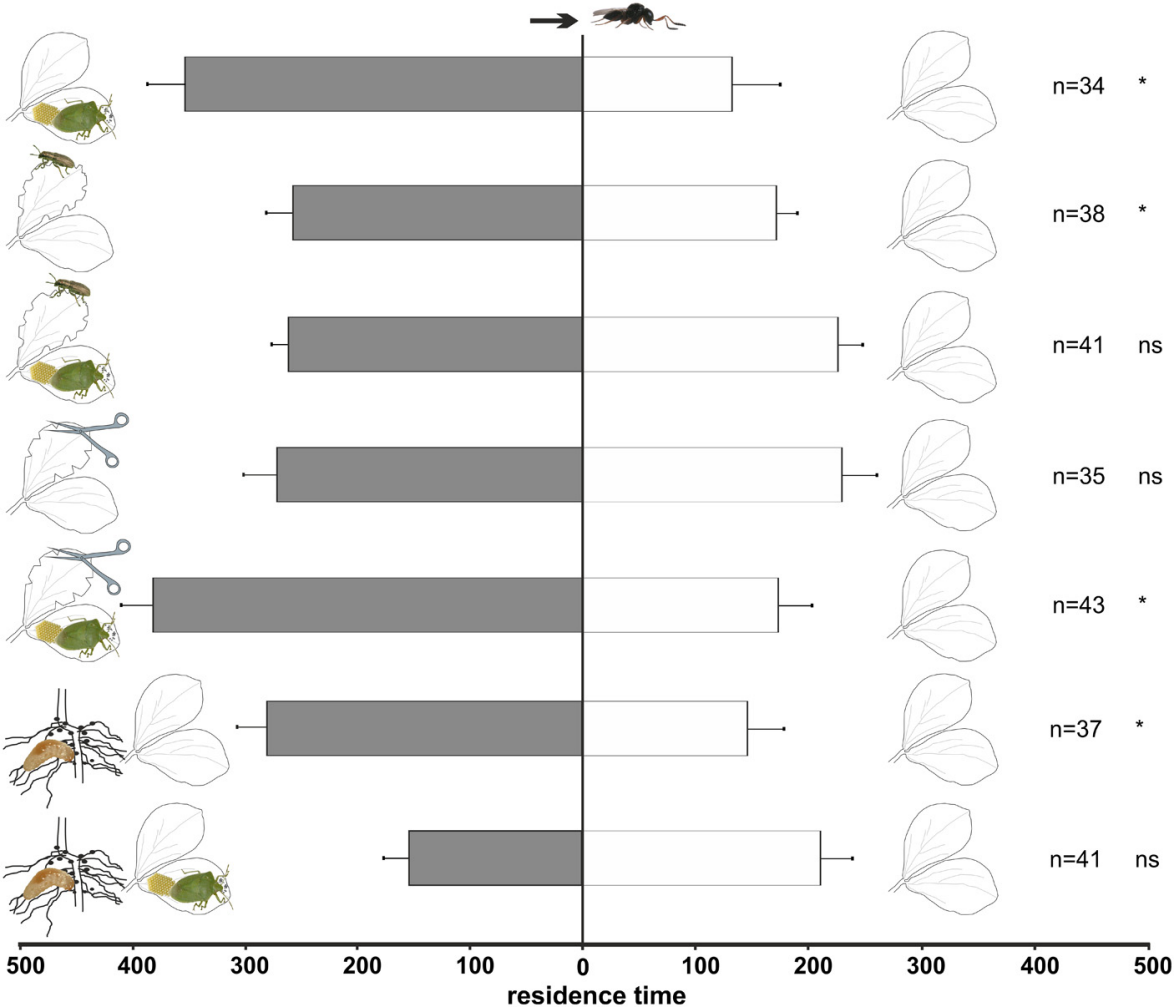
**Response of naïve *Trissolcus basalis* females in a Y-tube olfactometer to volatiles from *V. faba* plants subjected to above-ground and below-ground treatments *versus* healthy plants.** Plant treatments: *N. viridula* feeding and oviposition; *S. lineatus* adult leaf-feeding; *N. viridula* feeding and oviposition and *S. lineatus* adult leaf-feeding; mechanical damage; *N. viridula* feeding and oviposition and mechanical damage; *S. lineatus* larvae root-nodules feeding and above- + below-ground treatment (*N. viridula* feeding and oviposition and *S. lineatus* larvae root-nodules feeding). *n* = number of replicates. Bars represent mean (± SEM) of the time spent by wasp females in each arm over an observation period of 600 s (ns = not significant; * = *P* < 0.05).

Naïve *T. basalis* females (**Figure 2**) were significantly attracted to volatiles emitted by plants damaged by *N. viridula* feeding and oviposition (*t* = 4.75; df = 33; *p* < 0.001), and by plants damaged by leaf-feeding by *S. lineatus* adults (*t* = -2.13; df = 37; *p* = 0.040) compared to undamaged control plants. Although, a sensitivity of *S. lineatus* treated-plants to clip cages, not present in controls, cannot be excluded. However, these differences disappeared when plants were damaged by both *N. viridula* feeding and oviposition, and *S. lineatus* leaf-feeding, with these plants being equally attractive to controls (*t* = -0.87; df = 40; *p* = 0.389). The simple mechanical damage did not stimulate a significant response from naïve wasps compared to undamaged plants (*t* = 0.90; df = 34; *p* = 0.375), whereas the combination of *N. viridula* feeding and oviposition plus mechanical damage did (*t* = -3.50; df = 42; *p* = 0.001).

**FIGURE 3:**
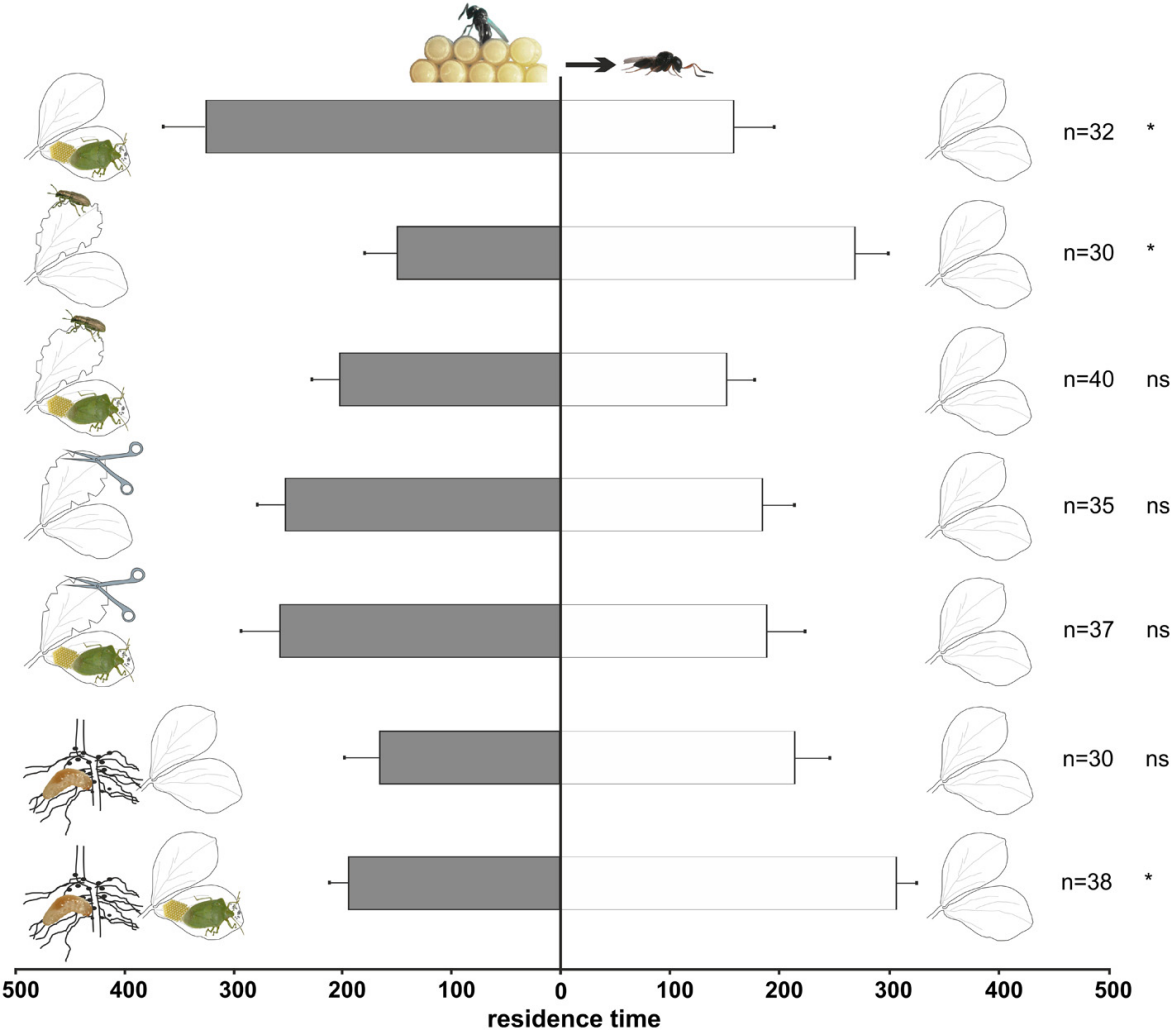
**Response of experienced *T. basalis* females in a Y-tube olfactometer to volatiles from *V. faba* plants subjected to above-ground and below-ground treatments *versus* healthy plants.** Plant treatments: *N. viridula* feeding and oviposition; *S. lineatus* adult leaf-feeding; *N. viridula* feeding and oviposition and *S. lineatus* adult leaf-feeding; mechanical damage; *N. viridula* feeding and oviposition and mechanical damage; *S. lineatus* larvae root-nodules feeding and above- + below-ground treatment (*N. viridula* feeding and oviposition and *S. lineatus* larvae root-nodules feeding). *n* = number of replicates. Bars represent mean (± SEM) of the time spent by wasp females in each arm over an observation period of 600 s (ns = not significant; * = *P* < 0.05).

Experienced female wasps (**Figure 3**) also showed a significant preference for volatiles released by plants with *N. viridula* feeding and oviposition compared to controls (*t* = -2.4; df = 30; *p* = 0.022). However, in contrast to naïve wasps, experienced wasps preferred the odors of undamaged plants to the odors from plants damaged by *S. lineatus* adult leaf-feeding (*t* = -2.33; df = 29; *p* = 0.027). No significant choice was displayed by experienced females when presented with the other above-ground treatments (*N. viridula* feeding and oviposition and *S. lineatus* adult leaf-feeding: *t* = -1.12; df = 39; *p* = 0.27; mechanical damage: *t* = -110.90; df = 34; *p* = 0.28; *N. viridula* feeding and oviposition and mechanical damage: *t* = -0.95; df = 36; *p* = 0.35).

#### Below-ground treatments

Naïve wasps were significantly attracted to volatiles emitted by plants damaged by *S. lineatus* larvae feeding on root nodules (*t* = 2.11; df = 36, *p* = 0.042; **Figure 2**) compared to controls, whereas experienced females did not discriminate between the treatment and control (*t* = 0.68; df = 29; *p* = 0.50; **Figure 3**).

#### Above- + below-ground treatments

Naïve parasitoids did not discriminate between volatiles emitted by plants damaged by *N. viridula* feeding and oviposition plus *S. lineatus* larval damage to root nodules *vs.* healthy plants (*t* = 1.31; df = 40; *p* = 0.20; **Figure 2**). In contrast, experienced parasitoids significantly preferred volatiles released by healthy plants to volatiles emitted by plants with *N. viridula* feeding and oviposition plus *S. lineatus* larval feeding on root nodules (*t* = 2.06; df = 38; *p* = 0.046; **Figure 3**).

### PLANT VOLATILES ANALYSIS

**FIGURE 4:**
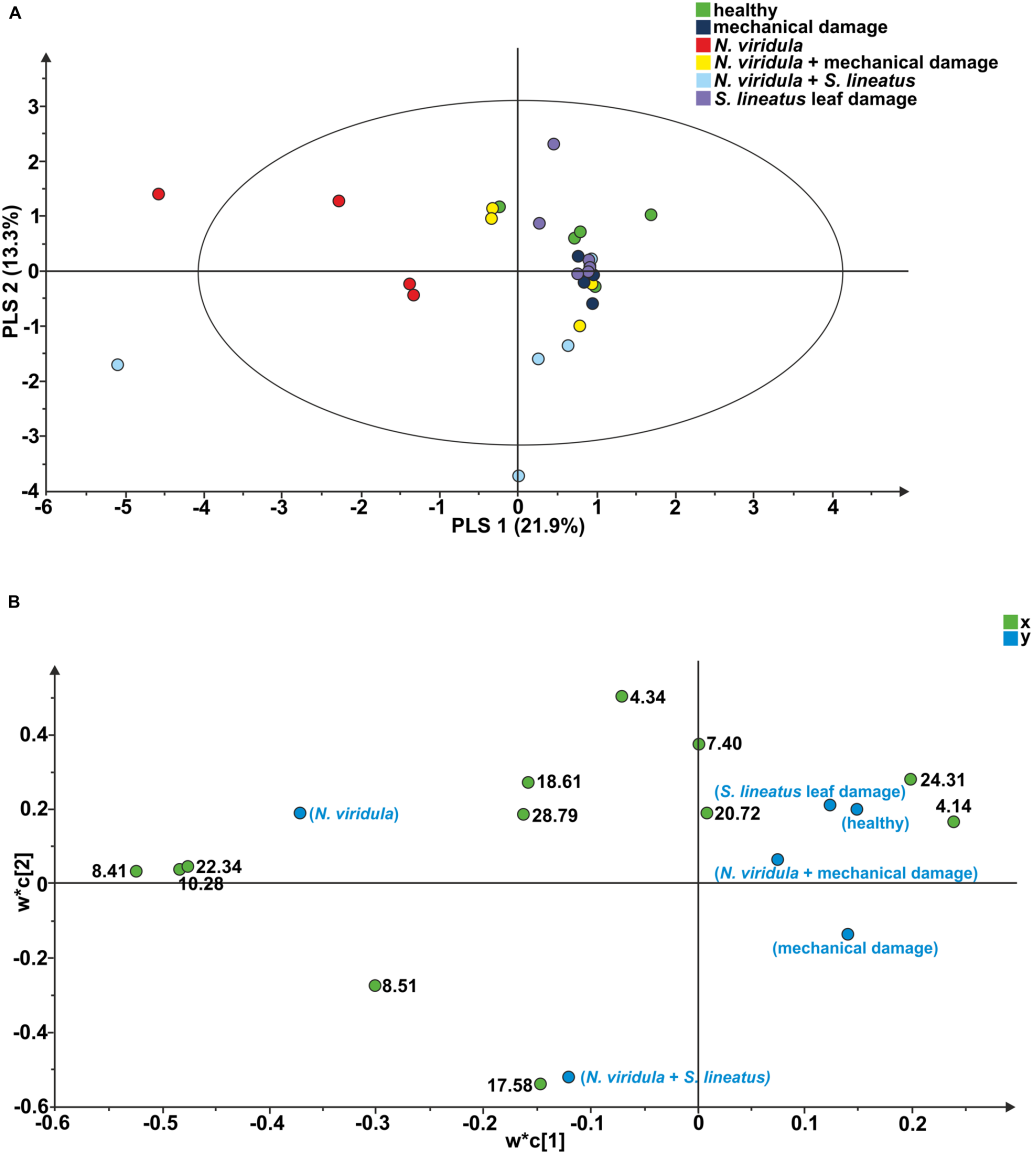
**Projection to latent structures discriminant analysis (PLS-DA) comparison of the volatile compounds emitted by individual *V. faba* plants. (A)** Score plot of the samples, with the percentage of explained variation in parentheses. The PLS-DA resulted in a model with two significant principal components (PCs). The ellipse defines the Hotelling’s T2 confidence region (95%). **(B)** Loading plot of the first two components of the PLS-DA, showing the contribution of each of the compounds toward the model. Numbers refer to the retention time of volatile compounds.

Twelve compounds were detected in the analyses of odors collected from *V. faba* plants. A PLS-DA comparison including samples of healthy plants and all above-ground treatments resulted in a model with two significant principal components (PCs; *R^2^*X = 0.219; *R^2^*Y = 0.135; *Q^2^* = -0.095; **Figure 4A**). In particular, the PLS-DA separated the plants subjected to *N. viridula* damage and the plants subjected to *N. viridula* + *S. lineatus* damage. Examination of the loading plot showed that a group of five compounds contributed the most to explaining the variation in the model (**Figure 4B**). These compounds have the following retention time (min) and corresponding VIP values (variable importance for the projection): (1) = 17.58, 1.37; (2) = 4.34, 1.29; (3) = 8.41, 1.25; (4) = 10.28, 1.16; (5) = 22.34, 1.15.

## DISCUSSION

In this study we demonstrated that, under our experimental conditions, a non-host herbivore species that feeds on both above- and below-ground plant parts can alter the responses of an egg parasitoid toward OIPVs. However, it is important to keep in mind that damage inflicted by non-host herbivores can vary in terms of intensity and duration, and that such factors can also affect plant responses as well as parasitoid foraging behavior (Ponzio et al., 2014). Previous investigations showed that the egg parasitoid *T. basalis* is attracted to OIPVs emitted by *V. faba* plants as a consequence of combination of egg deposition and feeding activity of the host *N. viridula* (Colazza et al., 2004a,b). However, the attraction of *T. basalis* to *V. faba* plants infested with *N. viridula* is eliminated when the plants are also attacked by *S. lineatus*, regardless of whether non-host infestation occurs on leaves or on roots. Studies on larval parasitoids have also demonstrated that below-ground herbivore species can disrupt infochemical networks. For example, Soler et al. (2007) provided evidence that the foraging behavior of *Cotesia glomerata* (L.), a larval parasitoid of *Pieris brassicae* (L.), can be affected by the below-ground herbivore *Delia radicum* (L.) through changes in the host plant (*Brassica nigra*) odor blend. Similarly, the congeneric *C. marginiventris* (Cresson) prefers odors emitted by host-infested plants over plants infested with both host and non-host root herbivores (Rasmann and Turlings, 2007).

In the case of dual above-ground stresses, naïve *T. basalis* were still attracted to odors emitted by plants damaged with a combination of *N. viridula* feeding and oviposition and mechanical damage, but naïve wasps were not attracted to plants suffering dual above-ground herbivore attacks (*N. viridula* feeding and oviposition + *S. lineatus* adult feeding). Plants are able to sense touch, feeding and oviposition activity of herbivore insects (Hilker and Meiners, 2010). However, even if it cannot be completely ruled out that plants can also sense the pressure induced by clip cages used in *S. lineatus* treatments, our preliminary results (Cusumano and Salerno personal observations) and previous investigations (Guerrieri et al., 1999) both suggest that clip cages have a negligible effect in the context of this study. The results discussed above suggest instead that *S. lineatus* oral secretions or damage patterns could be involved in altering the blend of induced volatiles. Indeed, many elicitors that plants use to activate indirect defense mechanisms have been identified in the oral secretions of insects that come in contact with plant tissues during feeding (Bonaventure, 2012). Oral secretions can also contain microorganisms that could potentially trigger plant responses (Zhu et al., 2014). The role of herbivore-associated microorganisms in plant defenses is an emerging and poorly understood area of plant-insect interactions, making it difficult to speculate about whether microorganisms are involved in our study system. Further investigations should be conducted to screen for the presence of microorganisms in *S. lineatus* oral secretions. Among the others factors that could explain our results, we doubt that differential patterns of herbivory played a major role, since mechanical damage was performed by carefully mimicking the damage inflicted by *S. lineatus* adults. PLS-DA analysis of the odor blends from the different treatments supports our behavioral data, with significant changes to odor profiles of *V. faba* plants as a consequence of single or dual herbivore attack. In previous studies of dual above-ground herbivore attack, natural enemy attraction was either disrupted, unaffected, or even enhanced by dually infested plants, indicating that the effect of multiple herbivore attack on HIPVs emissions is variable (Shiojiri et al., 2001; Agbogba and Powell, 2007; Moayeri et al., 2007; de Boer et al., 2008; Erb et al., 2010; Bukovinszky et al., 2012; de Rijk et al., 2013; Ponzio et al., 2014).

Interestingly, under our experimental conditions, *V. faba* damage inflicted either by *S. lineatus* adults or larvae had a similar disruptive effect on attraction of naïve *T. basalis*. This suggests that the damage caused by larvae and adults of *S. lineatus*, both of which are chewing insects even though they feed on different parts of the plant, may cause similar responses in the plant, which in turn may affect the feeding and oviposition activity of piercing-sucking insects. Considering previous studies on other model systems (Moran and Thompson, 2001; Thaler, 2002; Kempema et al., 2007; Zarate et al., 2007; Smith et al., 2009; Ode, 2013; Reymond, 2013) one could hypothesize that the disrupting effect of *S. lineatus* on *T. basalis* attraction is due to cross-talk between JA and SA pathways. So far, phytohormonal consequences of egg deposition have been investigated only in model systems of lepidopteran herbivores associated with brassicaceous plants, whereas nothing is known in other systems. Consequently, to confirm our hypothesis, further research on phytohormonal signaling pathways in response to feeding and oviposition activities of piercing-sucking insects is required.

In our study, an oviposition experience affected the response of *T. basalis* females to plant volatiles in a Y-tube olfactometer. It has often been suggested that learning can be adaptive for egg parasitoids when foraging for hosts in complex and dynamic environments (Fatouros et al., 2008; Colazza et al., 2010; Cusumano et al., 2012). Learning appears to be partially adaptive in our study as well, especially considering that volatiles induced in *V. faba* plants infested only with *S. lineatus* adults or larvae attracted naïve *T. basalis* females. For the parasitoids, attraction to *S. lineatus-*induced volatiles may be costly in terms of reproduction, because they would waste time searching on plants where there were no hosts present. This negative effect could be particularly severe for *T. basalis* given that it occurs twice during the *V. faba* growing season, considering the life history traits of univoltine species like *S. lineatus*. In fact, above-ground attacks occur early in the growing season whereas below-ground attacks occur later when the developing larvae feed on the roots. Such temporal dynamics of non-host herbivore infestation could considerably extend the temporal window of disturbance and consequently decrease the efficiency of host location by *T. basalis*. Oviposition experience on *N. viridula* eggs laid on *V. faba* leaves changed the behavioral responses of parasitoids so that they were no longer attracted to plants infested with *S. lineatus* adults or larvae, nor were they attracted to plants infested with both *N. viridula* and *S. lineatus*. Furthermore, the response showed by experienced *T. basalis* was not straightforward because wasps were not attracted to mechanically damaged plants that were also infested with *N. viridula*. Overall, the divergence of *T. basalis* behavior between naïve and experienced wasps suggests that the wasps are using associative learning to optimize their foraging efficiency (Steidle and Van Loon, 2003; Hoedjes et al., 2011; Gols et al., 2012). The role of learning in multitrophic systems has been investigated in few other case studies; for instance, the larval parasitoid *C. marginiventris* preferred HIPVs induced by its host *Spodoptera littoralis* (Boisduval), but after oviposition experience while exposed to maize plants infested with the host *S. littoralis* and the non–host *Diabrotica virgifera virgifera* (Leconte), the parasitoid preferred HIPV blends of maize induced by both herbivores (Rasmann and Turlings, 2007) All these results emphasize the need to control the pre-assay experience of egg parasitoids when evaluating their responses to induced plant volatiles, when the plants are being attacked by different combinations of herbivore species.

In summary, the present study investigated the effects of an above- and below-ground non-host herbivore on attraction of an egg parasitoid to plant volatiles induced by feeding and oviposition of its host. Our results demonstrated that attraction of this wasp was disrupted by both larvae and adults of *S. lineatus* when foraging for *N. viridula* eggs laid on *V. faba* plants. Further studies will focus on the identification of the volatile compounds emitted by *V. faba* plants that are attacked individually or concurrently by *N. viridula* and *S. lineatus* in order to identify the blend of compounds that play a role in egg parasitoid recruitment, and how that blend is altered or disrupted by *S. lineatus* feeding.

## AUTHOR CONTRIBUTIONS

Stefano Colazza, Ezio Peri, Antonino Cusumano, Gianandrea Salerno, Eric Conti, and Francesca Frati conceived and designed the experiments; Rihem Moujahed, Gianandrea Salerno, Antonino Cusumano and Francesca Frati performed the experiments and analyzed the data; all authors interpreted results, drafted and revised the article.

## Conflict of Interest Statement

The authors declare that the research was conducted in the absence of any commercial or financial relationships that could be construed as a potential conflict of interest.

## References

[B1] Agbogba B. C. Powell W. (2007). Effect of the presence of a non host herbivore on the response of the aphid parasitoid *Diaeretiella rapae* to host-infested cabbage plants. *J. Chem. Ecol.* 33 2229–2235 10.1007/s10886-007-9379-x 17968626

[B2] Bardner R. (1983). “Pests of *Vicia faba L*. other than aphids and nematodes,” in *The Faba Bean (Vicia faba L.): A Basis for Improvement* ed.Hebblethwaite P. D. (London: Butterworths) 371–390.

[B3] Bonaventure G. (2012). Perception of insect feeding by plants. *Plant Biol.* 14 872–880 10.1111/j.1438-8677.2012.00650.x 22957774

[B4] Bruessow F. Gouhier-Darimont C. Buchala A. Metraux J. P. Reymond P. (2010). Insect eggs suppress plant defence against chewing herbivores. *Plant J.* 62 876–885 10.1111/j.1365-313X.2010.04200.x 20230509

[B5] Bukovinszky T. Poelman E. H. Kamp A. Hemerik L. Prekatsakis G. Dicke M. (2012). Plants under multiple herbivory: consequences for parasitoid search behaviour and foraging efficiency. *Anim. Behav.* 83 501–509 10.1016/j.anbehav.2011.11.027

[B6] Colazza S. Fucarino A. Peri E. Salerno G. Conti E. Bin F. (2004a). Insect oviposition induces volatile emission in herbaceous plants that attracts egg parasitoids. *J. Exp. Biol.* 207 47–53 10.1242/jeb.00732 14638832

[B7] Colazza S. McElfresh J. S. Millar J. G. (2004b). Identification of volatile synomones, induced by *Nezara viridula* feeding and oviposition on Bean spp: That attract the egg parasitoid *Trissolcus basalis*. *J. Chem. Ecol.* 30 945–964 10.1023/B:JOEC.0000028460.70584.d1 15274441

[B8] Colazza S. Peri E. Salerno G. Conti E. (2010). “Host searching by egg parasitoids: exploitation of host chemical cues,” in *Egg Parasitoids in Agroecosystems with Emphasis on Trichogramma* eds Parra J. R. P. Consoli F. L. Zucchi R. A. (New York: Springer)97–147.

[B9] Colazza S. Salerno G. Wajnberg E. (1999). Volatile and contact chemicals released by *Nezara viridula* (Heteroptera:Pentatomidae) have a kairomonal effect on the egg parasitoid *Trissolcus basalis* (Hymenoptera:Scelionidae). *Biol. Control* 16 310–317 10.1006/bcon.1999.0763

[B10] Conti E. Colazza S. (2012). Chemical ecology of egg parasitoids associated with true bugs. *Psyche* 2012:651015 10.1155/2012/651015

[B11] Corre-Hellou G. Crozat Y. (2005). N2 fixation and N supply in organic pea (*Pisum sativum* L.) cropping systems as affected by weeds and peaweevil (*Sitona lineatus* L.). *Eur. J. Agron.* 22 449–458 10.1016/j.eja.2004.05.005

[B12] Cusumano A. Peri E. Vinson S. B. Colazza S. (2012). Interspecific extrinsic and intrinsic competitive interactions in egg parasitoids. *Biocontrol* 57 719–734 10.1007/s10526-012-9451-5

[B13] de Boer J. G. Hordijk C. A. Posthumus M. A. Dicke M. (2008). Prey and non-prey arthropods sharing a host plant: effects on induced volatile emission and predator attraction. *J. Chem. Ecol.* 34 281–290 10.1007/s10886-007-9405-z 18185960 PMC2266969

[B14] de Rijk M. Dicke M. Poelman E. H. (2013). Foraging behaviour by parasitoids in multiherbivore communities. *Anim. Behav.* 85 1517–1528 10.1016/j.anbehav.2013.03.034

[B15] Dicke M. (2009). Behavioural and community ecology of plants that cry for help. *Plant Cell Environ.* 32 654–665 10.1111/j.1365-3040.2008.01913.x 19021885

[B16] Dicke M. Baldwin I. T. (2010). The evolutionary context for herbivore-induced plant volatiles: beyond the ‘cry for help’. *Trends Plant Sci.* 15 167–175 10.1016/j.tplants.2009.12.002 20047849

[B17] Erb M. Foresti N. Turlings T. (2010). A tritrophic signal that attracts parasitoids to host-damaged plants withstands disruption by non-host herbivores. *BMC Plant Biol.* 10:247 10.1186/1471-2229-10-247 PMC3095329 21078181

[B18] Fatouros N. E. Dicke M. Mumm R. Meiners T. Hilker M. (2008). Foraging behavior of egg parasitoids exploiting chemical information. *Behav. Ecol.* 19 677–689 10.1093/beheco/arn011

[B19] Fatouros N. E. Lucas-Barbosa D. Weldegergis B. T. Pashalidou F. G. van Loon J. J. A. Dicke M. (2012). Plant volatiles induced by herbivore egg deposition affect insects of different trophic levels. *PLoS ONE* 7:e43607 10.1371/journal.pone.0043607 PMC3422343 22912893

[B20] Gols R. Veenemans C. Potting R. P. J. Smid H. M. Dicke M. Harvey J. A. (2012). A variation in the specificity of plant volatiles and their use by a specialist and a generalist parasitoid. *Anim. Behav.* 83 1231–1242 10.1016/j.anbehav.2012.02.015

[B21] Guerrieri E. Poppy G. M. Powell W. Tremblay E. Pennacchio F. (1999). Induction and systemic release of herbivore-induced plant volatiles mediating in-flight orientation of Aphidius ervi. *J. Chem. Ecol.* 25 1247–1261 10.1023/A:1020914506782

[B22] Hoebeke E. R. Wheeler A. G. J. R. (1985). *Sitona lineatus* (L.) the pea leaf weevil: first records in eastern north America (Coleoptera: Curcuclionidae). *Proc. Entomol. Soc. Wash.* 87 216–220.

[B23] Hoedjes K. M. Kruidhod H. M. Huigens M. E. Dicke M. Vet L. E. M. Smid H. M. (2011). Natural variation in learning rate and memory dynamics in parasitoid wasps: opportunities for converging ecology and neuroscience. *Proc. Biol. Sci.* 278 889–897 10.1098/rspb.2010.2199 21106587 PMC3049055

[B24] Hilker M. Meiners T. (2002). Induction of plant responses towards oviposition and feeding of herbivorous arthropods: a comparison. *Entomol. Exp. Appl.* 104 181–192 10.1046/j.1570-7458.2002.01005.x

[B25] Hilker M. Meiners T. (2010). How do plants “notice” attack by herbivorous arthropods? *Biol. Rev.* 85 267–280 10.1111/j.1469-185X.2009.00100.x 19961475

[B26] Johnson M. P. O’Keeffe L. E. (1981). Presence and possible assimilation of *Rhizobium leguminosarum* in the gut of pea leaf weevil, *Sitona lineatus*, larvae. *Entomol. Exp. Appl.* 29 103–108 10.1111/j.1570-7458.1981.tb03047.x

[B27] Kempema L. A. Cui X. Holzer F. M. Walling L. L. (2007). *Arabidopsis* transcriptome changes in response to phloem-feeding silver leaf whitefly nymphs. Similarities and distinctions in responses to aphids. *Plant Physiol.* 143 849–865 10.1104/pp.106.090662 17189325 PMC1803730

[B28] Kessler A. Baldwin I. T. (2001). Defensive function of herbivore-induced plant volatile emissions in nature. *Science* 291 2141–2144 10.1126/science.291.5511.2141 11251117

[B29] Kessler A. Heil M. (2011). Evolutionary ecology of plant defences. The multiple faces of indirect defences and their agents of natural selection. *Funct. Ecol.* 25 348–357 10.1111/j.1365-2435.2010.01818.x

[B30] Meiners T. Peri E. (2013). “Chemical ecology of insect parasitoids: essential elements for developing effective biological control programmes,” in *Chemical Ecology of Insect Parasitoids* eds Wajnberg E. Colazza S. (Oxford: Wiley-Blackwell) 193–224.

[B31] Moayeri H. R. S. Ashouri A. Poll L. Enkegaard A. (2007). Olfactory response of a predatory mirid to herbivore induced plant volatiles: multiple herbivory vs. single herbivory. *J. Appl. Entomol.* 131 326–332 10.1111/j.1439-0418.2007.01177.x

[B32] Moran P. J. Thompson G. A. (2001). Molecular responses to aphid feeding in *Arabidopsis* in relation to plant defense pathways. *Plant Physiol.* 125 1074–1085 10.1104/pp.125.2.1074 11161062 PMC64906

[B33] Nuessly G. S. Hentz M. G. Beiriger R. Scully B. T. (2004). Insects associated with faba bean *Vicia faba* (Fabales:Fabaceae), in southern Florida. *Fla. Entomol.* 87 204–211 10.1653/0015-4040(2004)087[0204:IAWFBV]2.0.CO;2

[B34] Ode P. J. (2013). “Plant defences and parasitoid chemical ecology,” in *Chemical Ecology of Insect Parasitoids*eds Wajnberg E. Colazza S. (Oxford:Wiley-Blackwell)11–28.

[B35] Peri E. Sole M. A. Wajnberg E. Colazza S. (2006). Effect of host kairomones and oviposition experience on the arrestment behavior of an egg parasitoid. *J. Exp. Biol.* 209 3629–3635 10.1242/jeb.02416 16943503

[B36] Ponzio C. Gols R. Weldegergis B. T. Dicke D. (2014). Caterpillar-induced plant volatiles remain a reliable signal for foraging wasps during dual attack with a plant pathogen or non-host insect herbivore. *Plant Cell Environ.* 37 1924–1935 10.1111/pce.12301 24697624

[B37] Rasmann S. Turlings T. C. J. (2007). Simultaneous feeding by aboveground and belowground herbivores attenuates plant-mediated attraction of their respective natural enemies. *Ecol. Lett.* 10 926–936 10.1111/j.1461-0248.2007.01084.x 17845293

[B38] Reymond P. (2013). Perception, signaling and molecular basis of oviposition-mediated plant responses. *Planta* 238 247–258 10.1007/s00425-013-1908-y 23748628 PMC3722449

[B39] Schoonhoven L. M. van Loon J. J. A. Dicke M. (2005). *Insect-Plant Biology.* Oxford: Oxford University Press.

[B40] Shiojiri K. Takabayashi J. Yano S. Takafuji A. (2001). Infochemically mediated tritrophic interactions webs on cabbage plants. *Popul. Ecol.* 43 23–29 10.1007/PL00012011

[B41] Smith J. L. De Moraes C. M. Mescher M. C. (2009). Jasmonate- and salicylate-mediated plant defense responses to insect herbivores, pathogens and parasitic plants. *Pest Manag. Sci.* 65 497–503 10.1002/ps.1714 19206090

[B42] Soler R. Bezemer T. M. Harvey J. A. (2013). “Chemical ecology of insect parasitoids in a multitrophic above- and below-ground context,” in *Chemical Ecology of Insect Parasitoids* eds Wajnberg E. Colazza S. (Oxford:Wiley-Blackwell) 64–85.

[B43] Soler R. Harvey J. A. Bezemer T. M. Kamp A. F. D. Vet L. E. M. van der Putten W. H. (2007). Root herbivores influence the behaviour of an aboveground parasitoid through changes in plant-volatile signals. *Oikos* 116 367–376 10.1111/j.0030-1299.2007.15501.x

[B44] Stam J. M. Kroes A. Li Y. Gols R. van Loon J. J. Poelman E. H. (2014). Plant interactions with multiple insect herbivores: from community to genes. *Plant Biol.* 65 689–713 10.1146/annurev-arplant-050213-035937 24313843

[B45] StatSoft (2001). *Statistica: Data Analysis Software System, Version* 7.1 Tulsa, OK: StatSoft.

[B46] Steidle J. L. M. Van Loon J. J. A. (2003). Dietary specialization and infochemical use in carnivorous arthropods: testing a concept. *Entomol. Exp. Appl.* 108 133–148 10.1046/j.1570-7458.2003.00080.x

[B47] Thaler J. S. (2002). Effect of jasmonate-induced plant responses on the natural enemies of herbivores. *J. Anim. Ecol.* 71 141–150 10.1046/j.0021-8790.2001.00586.x

[B48] van Emden H. F. Ball S. L. Rao M. R. (1988). “Pest, disease and weed problems in pea, lentil, faba bean and chickpea,” in *World Crops: Cool Season Food Legumes* ed.Summerfield R. J. (Dordrecht:Kluwer Academic Publishers), 519–534.

[B49] Zarate S. I. Kempema L. A. Walling L. L. (2007). Silverleaf whitefly induces salicylic acid defenses and suppresses effectual jasmonic acid defenses. *Plant Physiol.* 143 866–875 10.1104/pp.106.090035 17189328 PMC1803729

[B50] Zhu F. Poelman E. H. Dicke M. (2014). Insect herbivore-associated organisms affect plant responses to herbivory. *New Phytol.* 204 315–321 10.1111/nph.12886

